# Supplementary material for: Alveolar epithelial progenitor cells require Nkx2-1 to maintain progenitor-specific epigenomic state during lung homeostasis and regeneration
Source: Nat Commun. 2023 Dec 19;14:8452. doi: 10.1038/s41467-023-44184-0 (PMC10775890; doi:10.1038/s41467-023-44184-0)
Supplement: Supplementary file 1 — Supplemental Information [file 41467_2023_44184_MOESM1_ESM.pdf]

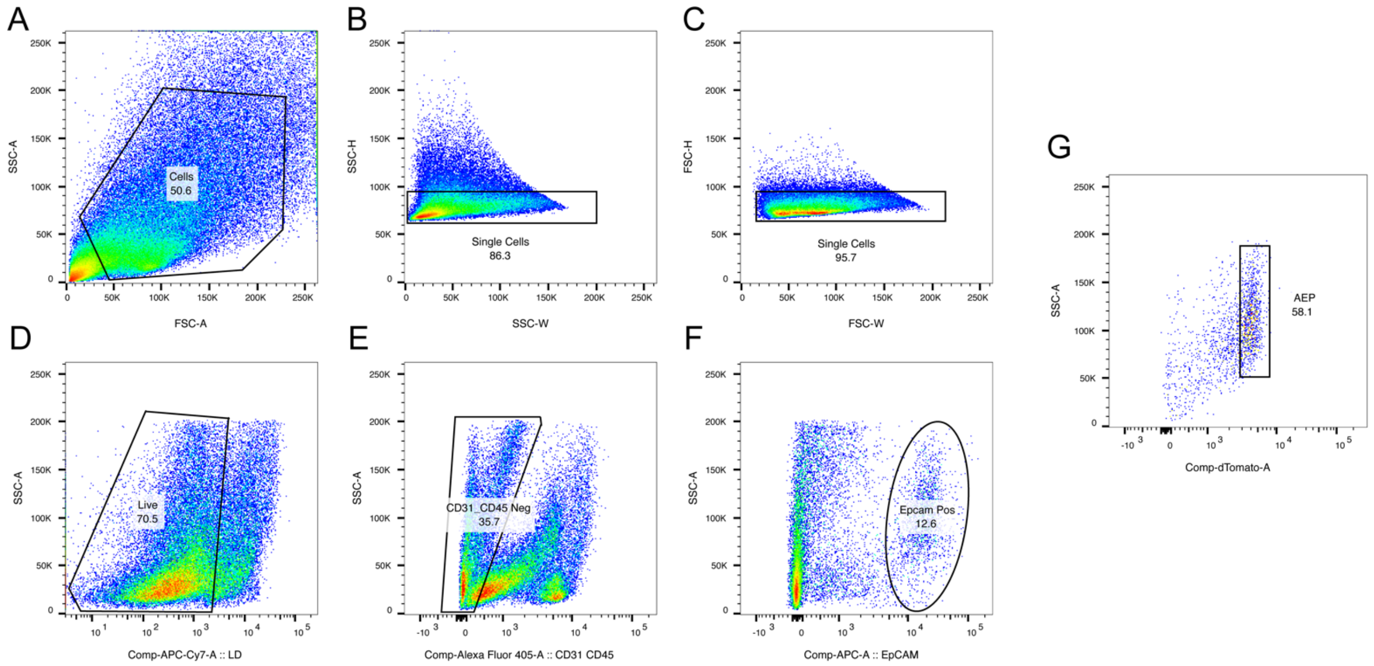

Supplemental Figure 1 | Sorting gates used to isolate Axin2-positive AT2 cells (AEPs) for use in organoids.
(A-F) Using the methods described in[3] and herein, cells were gated away from debris based on size **(A)**, then single cells were identified by SSC and FSC gating **(B, C)**. Live cells were identified by Live/Dead staining **(D)**, followed by removal of CD31- or CD45-positive cells in a dump channel **(E)**. Epithelial cells were identified by EpCAM expression **(F)**, and TdTomato-positive epithelium were sorted into complete SAGM media and used immediately for organoids (main figures) or single cell RNA sequencing (Supplemental Figure 5).

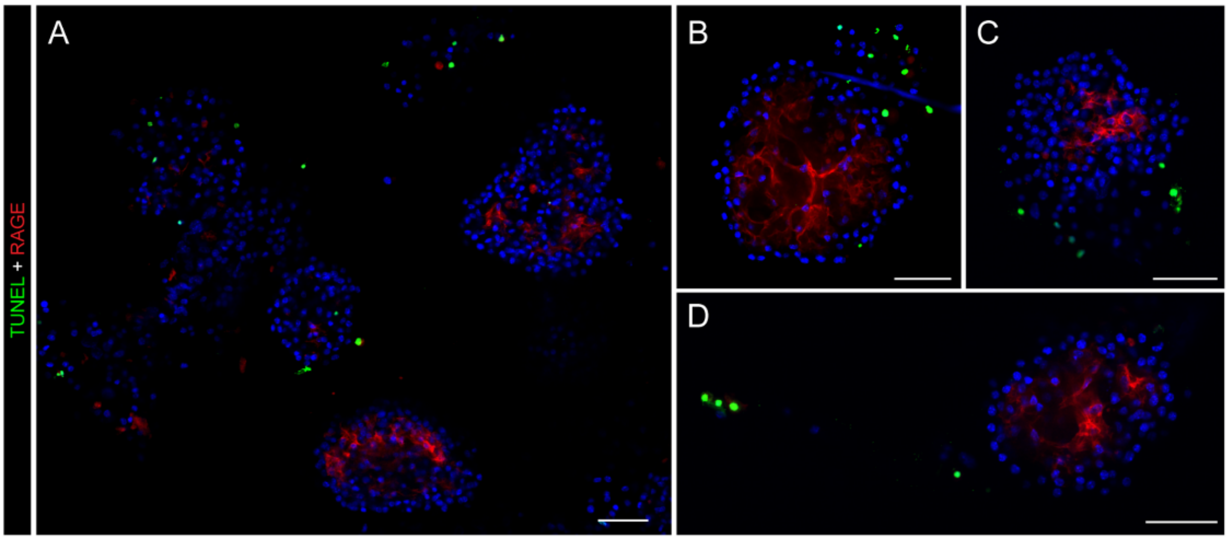

Supplemental Figure 2 | TUNEL$^+$ cells largely confined to cell clumps and debris outside organoids.
(A-D) Whole-mount immunofluorescence and Click-iT TUNEL staining showing lack of TUNEL+ cells within mature, healthy day 25 organoids. [*Scale bars = 50 μm*]; (*TUNEL = Terminal deoxynucleotidyl transferase dUTP nick end labeling [indicator of DNA strand breaks/apoptosis]; RAGE = Receptor for Advanced Glycation End-products [AT1 cell marker]*)

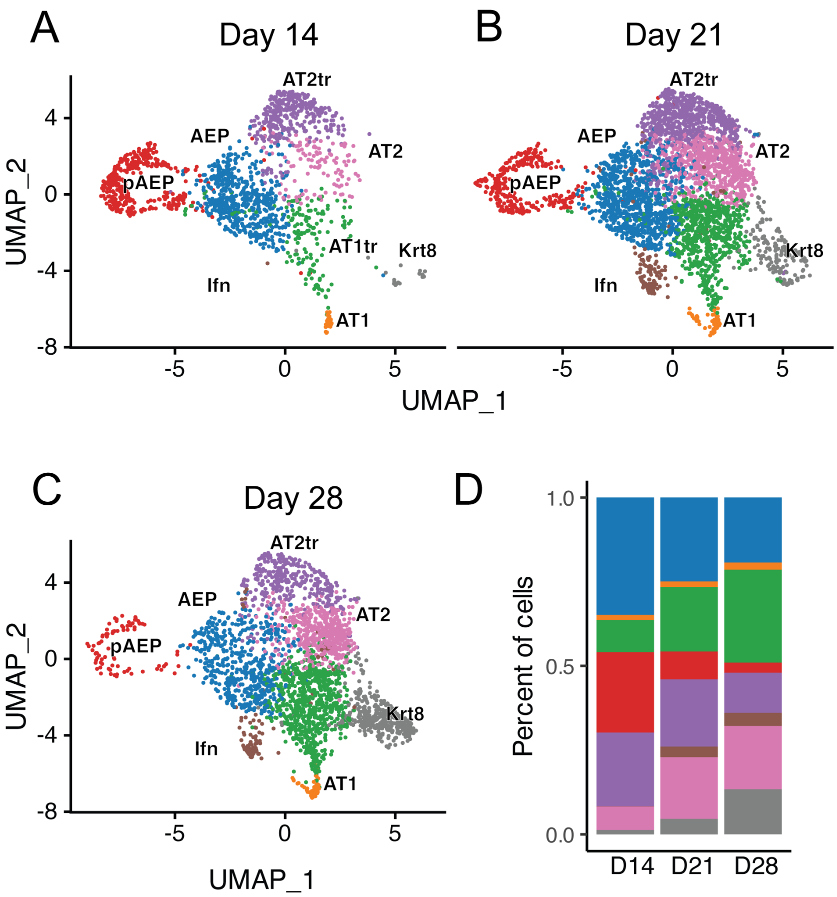

Supplemental Figure 3 | Evolution and contribution of epithelial cell states per timepoint in AEP-O.
(A-C) UMAP projections (left) demonstrating relative detected cells for each epithelial cell state at day 14 **(A)**, day 21 **(B)**, and 28 **(C)**. **(D)** Quantification of cell population abundance at each time point.

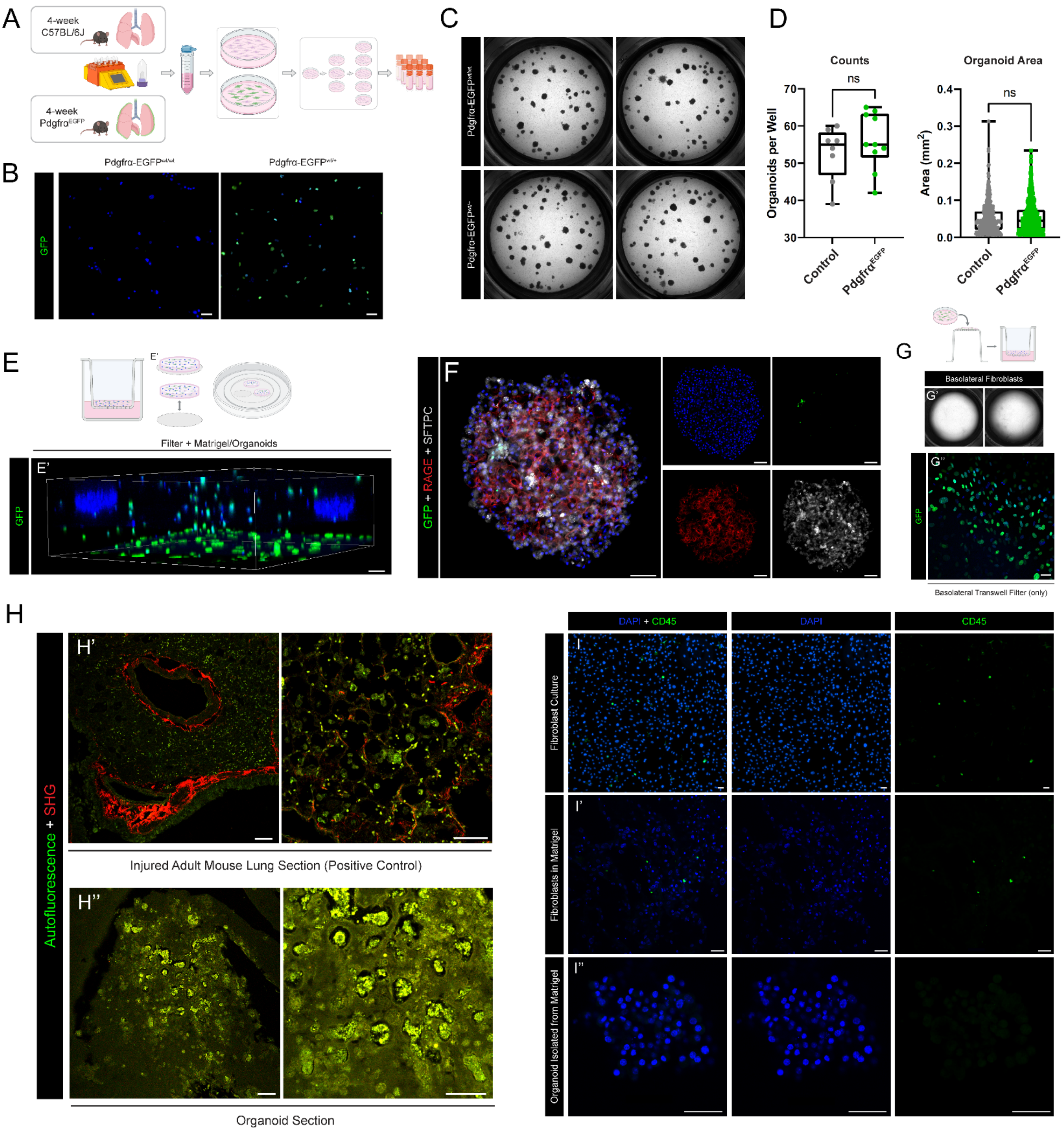

Supplemental Figure 4 | Localization of mesenchymal and immune cells in wells surrounding AEP-O.
(A) Generation of fibroblast stocks from control (C57BL/6J) and PDGFRα^{EGFP} mice. **(B)** Imaging of control and GFP fibroblast stocks at P2 (second passage). **(C)** Whole-well scans comparing day 35 AEP-derived organoids grown from control and PDGFRα^{EGFP} fibroblasts. **(D)** Quantification comparing counts and size of day 35 AEP-derived organoids grown from control and PDGFRα^{EGFP} fibroblasts. **(E)** Schematic of experimental set-up of live imaging; 3D reconstruction of live day 20 organoids and PDGFRα^{EGFP} fibroblasts stained with Hoechst; **(E')** 3D reconstruction of confocal z-stacks of Matrigel/organoids with the Transwell filter, showing the majority of GFP$^+$ fibroblasts are growing on the filter and not within organoids; **(F)** Whole-mount immunofluorescence of organoids grown with PDGFRα^{EGFP} fibroblasts showing lack of PDGFRα$^+$ cells within day 35 organoids. **(G)** Cultures with fibroblasts on the basolateral side of the filter and AEPs on the apical side of the filter do not grow organoids (G' – 31-day culture), although fibroblasts do persist on the basolateral side of the filter after 31 days **(G'')**. **(H)** Confocal imaging utilizing second harmonic generation to show lack of fibrillar collagen I and II in paraffin sections of organoids from long term culture **(H'')** (injured adult mouse lung section as positive control **[H']**). **(I-I'')** Immunofluorescence staining showing presence of contaminating immune (CD45$^+$) population (green) in fibroblast stocks **(I)**. Whole-mount immunofluorescence of day 28 Matrigel/fibroblast mixture showing presence of contaminating immune (CD45$^+$) population (green) outside organoids in Transwell cultures **(I')**. Whole-mount immunofluorescence of day 28 organoids showing lack of immune (CD45$^+$) population (green) inside organoids **(I'')**. (*ns = p > 0.05).* [*Scale bars = 50 μm*]. Source data are provided within the "Source Data" file. Schematics created with Biorender.com.

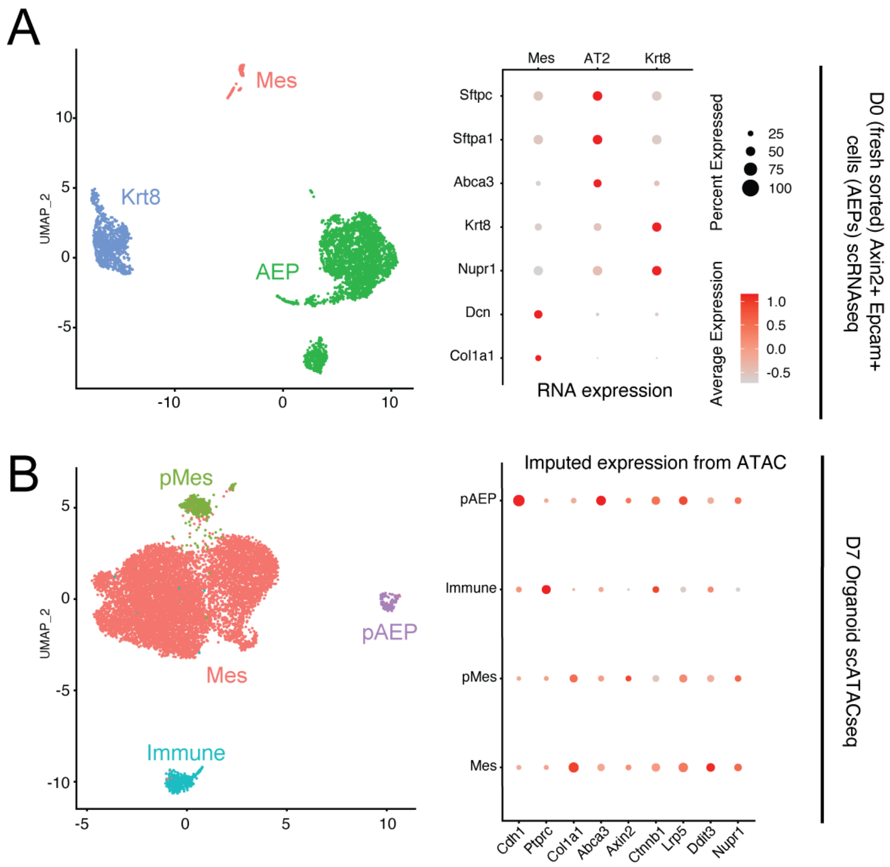

Supplemental Figure 5 | The sorted AEP fraction contains both stressed and unstressed cells at baseline, but organoids arise from AEP-like AT2 by day 7 of organoid culture.

(A) scRNAseq analysis of freshly sorted AEPs. Two epithelial populations are apparent with a small mesenchymal contaminant. Right panel shows marker genes for each cell population. **(B)** scATACseq of d7 AEP-O. The majority of cells at this stage are mesenchymal, with a small epithelial population which appears most similar to the pAEP state seen in late scRNAseq. Imputed gene expression from ATAC data is shown in right panel for marker gene identification. Low level expression of Krt8 markers is present, with higher level expression of AT2 genes such as Abca3 and AEP-enriched genes in the Wnt signaling pathway.

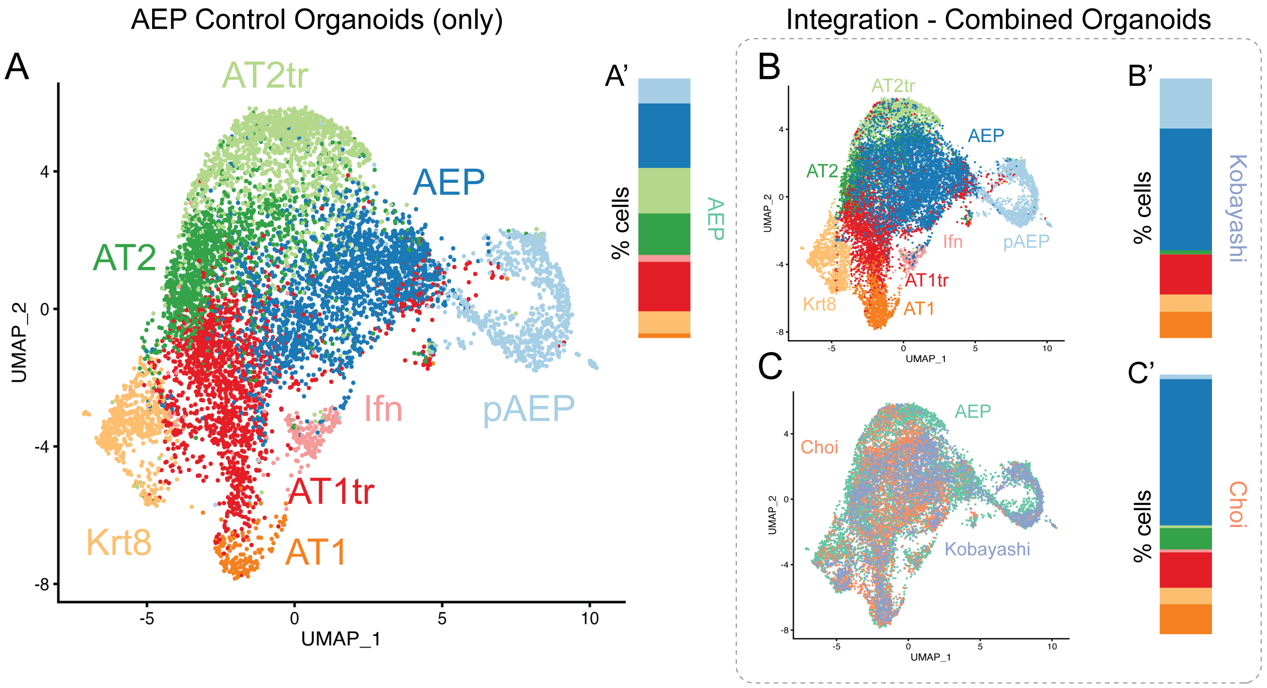

Supplemental Figure 6 | Comparison of AEP-derived organoids to publicly available scRNA data.

(A) UMAP of AEP organoids used as basis of integration and labels. **(B-C)** Integrated data from all three organoid datasets, labeled by cell type **(B)** or dataset of origin **(C)**. Composition of each organoid dataset is shown in **A-C'**.

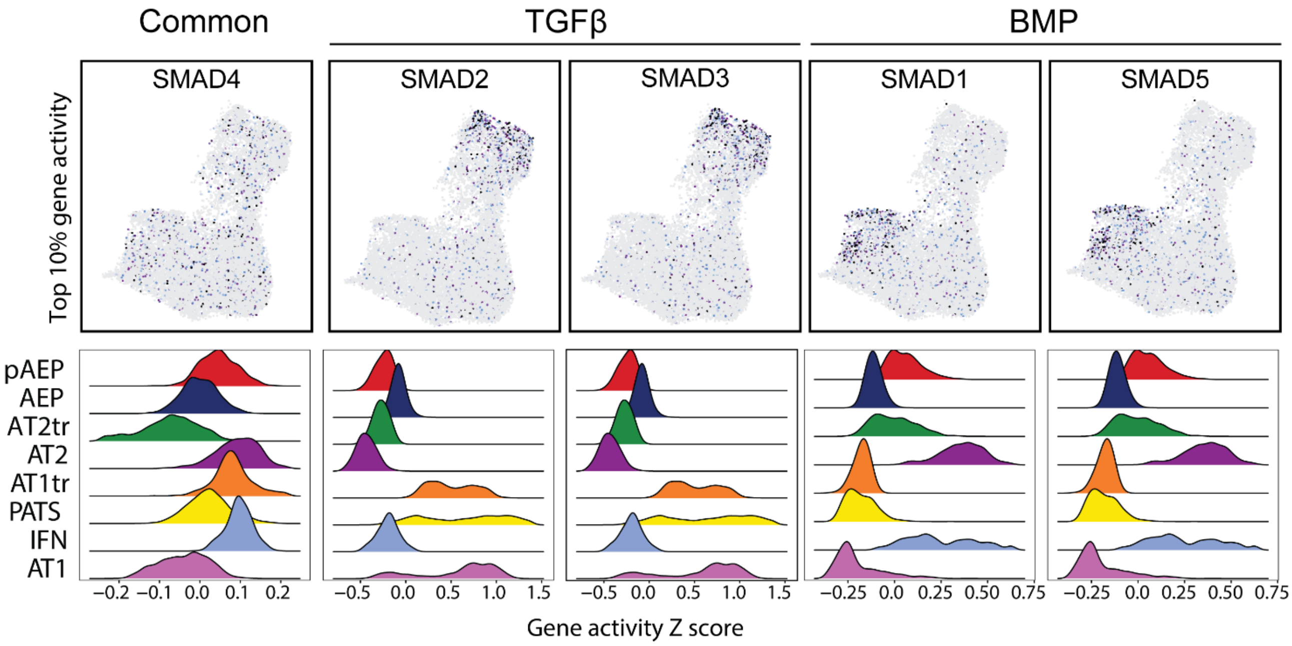

Supplemental Figure 7 | Transition of SMAD-regulated gene expression in AT2 to AT1 transitions in AEP-O.
Top row shows gene activity of SMAD target genes overlayed on scATACseq UMAP (compare to Figure 4A). Bottom row shows gene activity Z score of SMADs in each cell population in AEP-O; BMP signaling is predicted to be higher in AT2 cells, while TGFβ signaling predominates in AT1 cells.

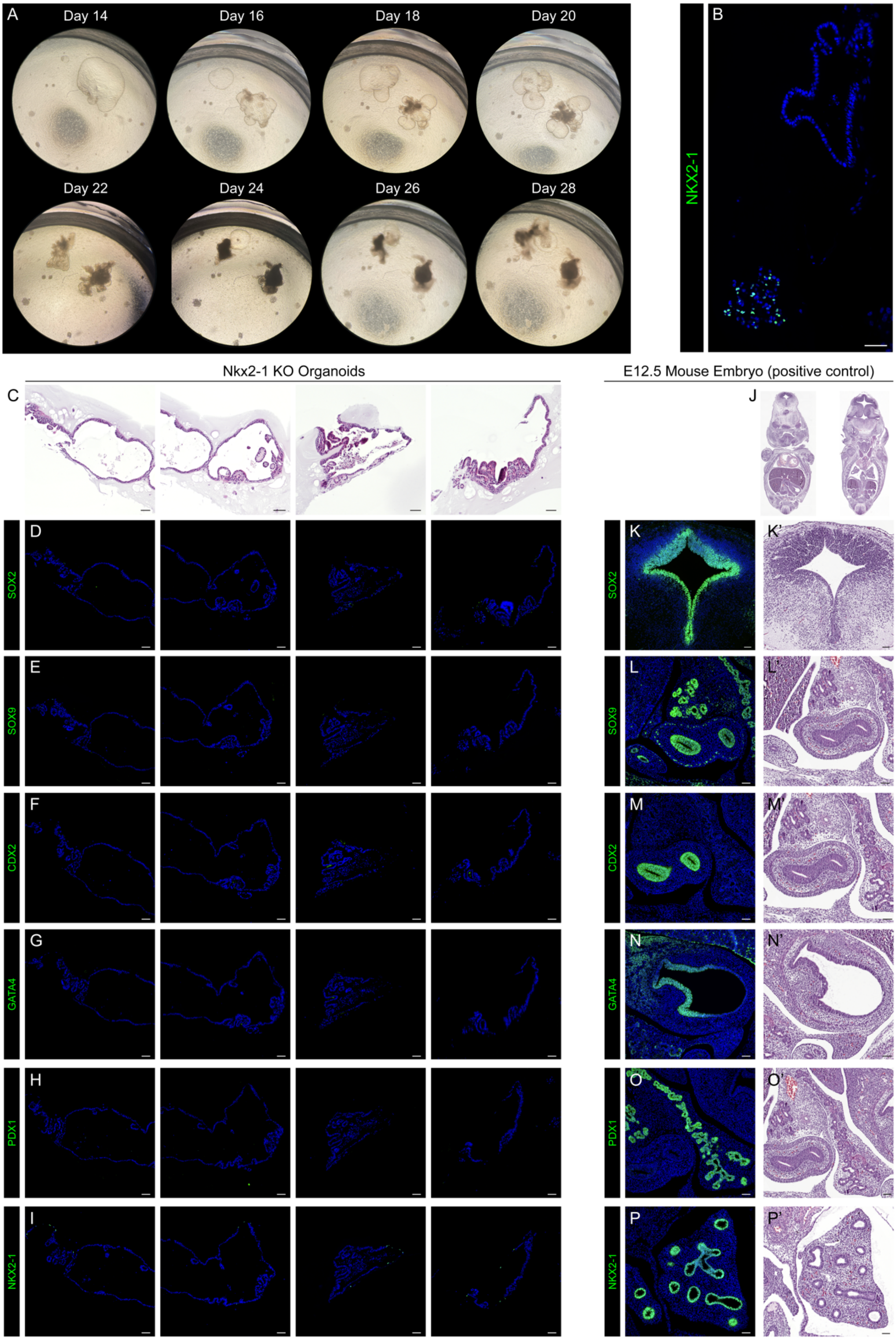

Supplemental Figure 8 | Nkx2-1 KO organoids lack substantial protein expression of canonical endoderm markers.

(A) Time series of Nkx2-1 KO organoids in culture from day 12 to day 28. **(B)** Immunofluorescence of paraffin section from Nkx2-1 organoid Transwells showing non-recombined (Nkx2-1^{+}) organoids adjacent to recombined (Nkx2-1^{-}) organoids with atypical morphology. **(C, J)** H&E of paraffin sections of Nkx2-1 KO organoids **(C)** and E12.5 mouse embryos **(J; K'-P')** used as positive controls for protein expression. Immunofluorescence of paraffin section from Nkx2-1 KO organoids **(D-I)** and E12.5 mouse embryos (positive controls; **K-P**) stained for canonical endodermal markers – Sox2 **(D, K)**, Sox9 **(E, L)**, Cdx2 **(F, M)**, Gata4 **(G, N)**, Pdx1 **(H, O)**, and Nkx2-1 **(I, P)**. [*Scale bars = 50 µm*].

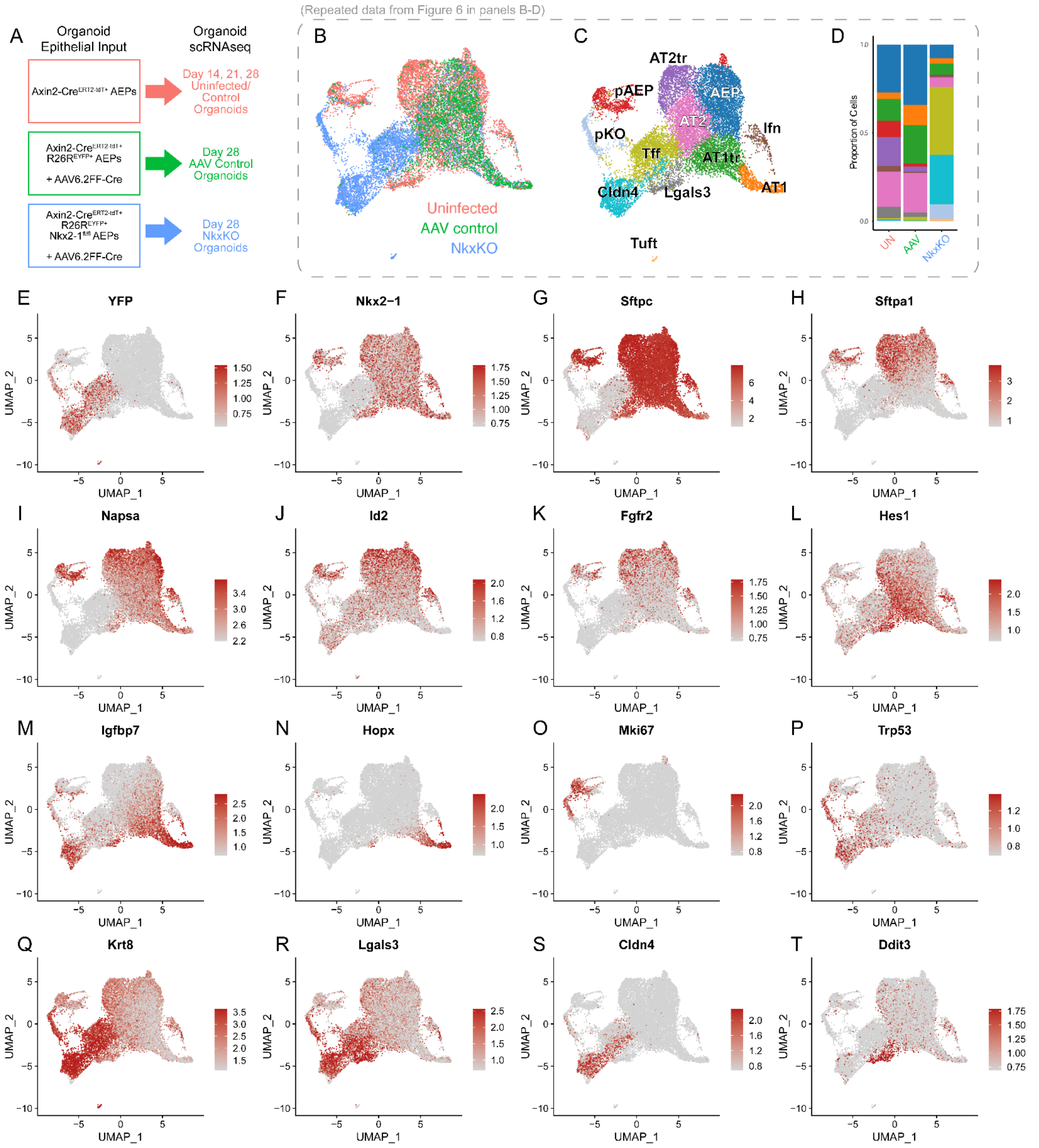

Supplemental Figure 9 | RNA Expression of Canonical Markers in Control (Uninfected), AAV Control, and Nkx2-1 KO AEP-derived Organoids.

(A) Overview of cell input used to generate AEP-O and subsequent timepoints for scRNAseq. **(B-D)** Repeated data from Figure 6K-M; Source **(B)**, cell clusters **(C)**, and proportion of cell type by experimental condition **(D)** for organoids outlined in **(A)**. **(E-T)** RNA expression of common markers of alveolar epithelial cell identity.

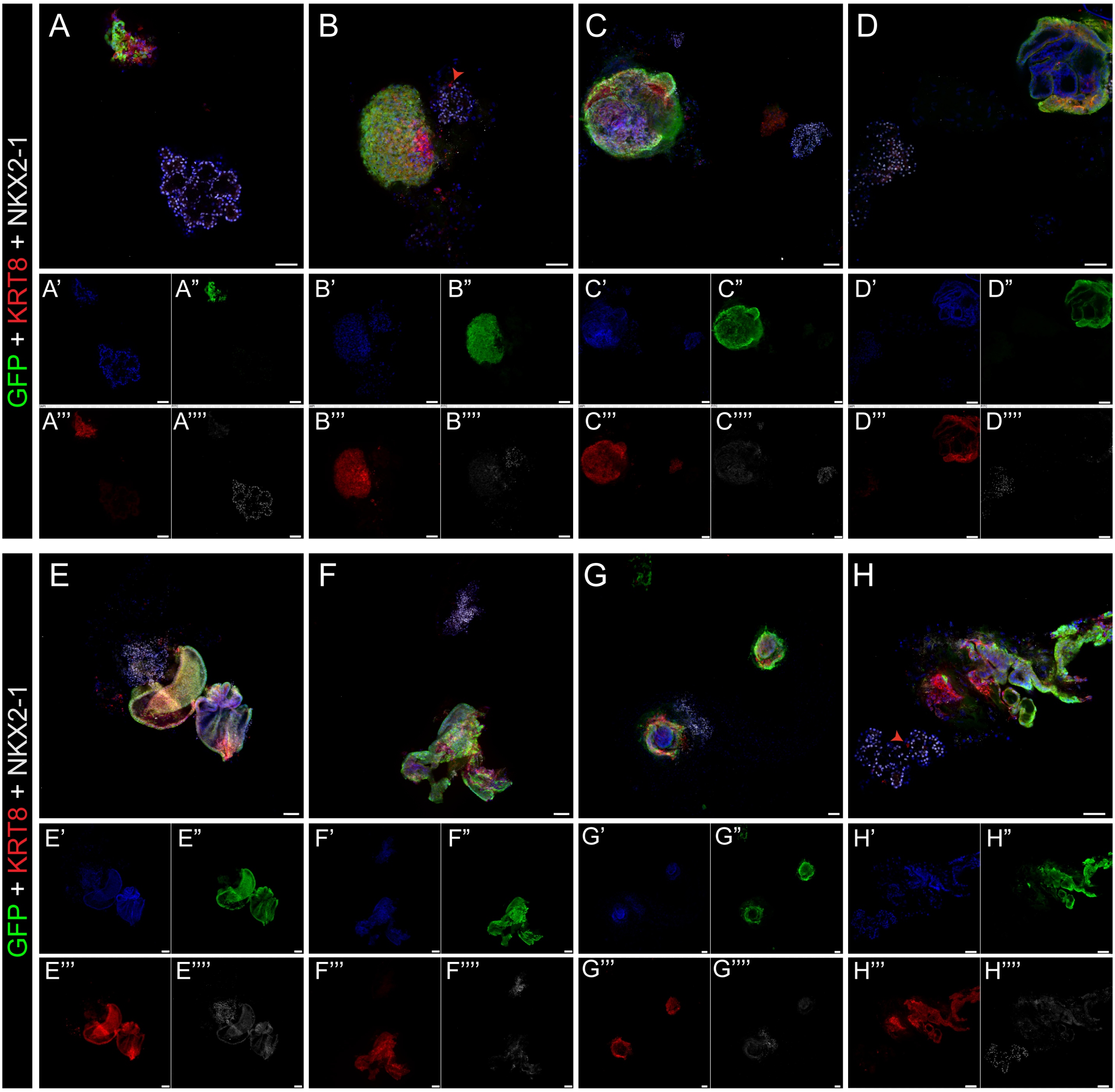

Supplemental Figure 10 | Morphology and Krt8 protein expression in Nkx2-1 KO compared to wild type organoids.
(A-H) Examples of GFP^{+} Nkx2-1^{KO} and adjacent WT organoids in whole mount IHC preparations from the same well of targeted organoids. Nkx2-1 KO organoids show high expression of GFP and Krt8, while adjacent WT organoids express Nkx2-1 and have an alveolar-like morphology. Red arrowheads in B and H highlight rare Krt8^{high} transitional cells found in WT organoids. Data represents N=3 distinct biological replicates. [*Scale bars = 50 μm*].

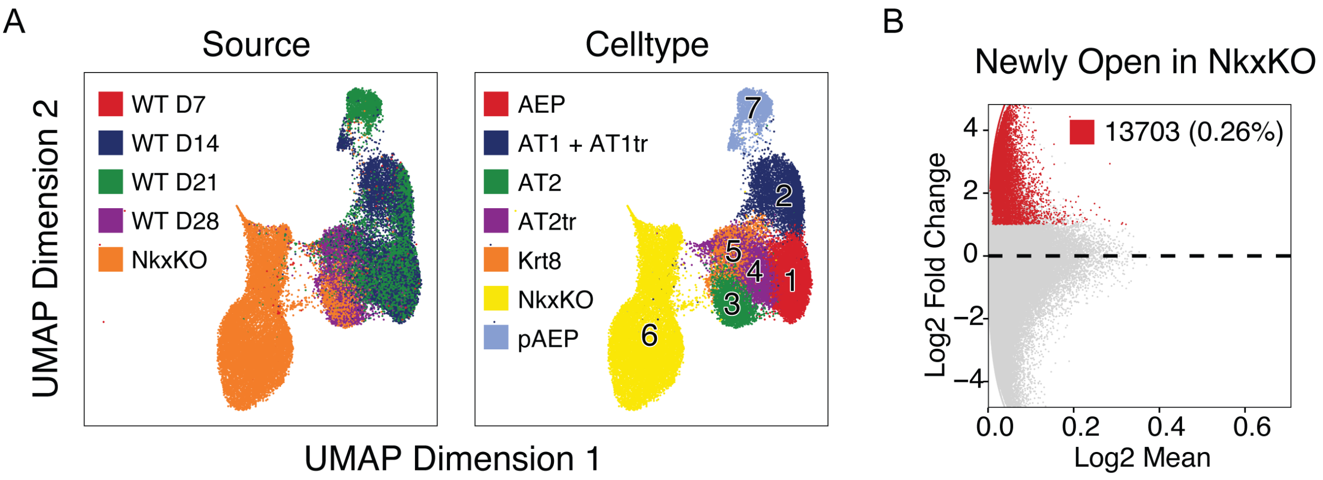

Supplemental Figure 11 | scATAC analysis of Nkx2-1 KO state *in vitro*.
(A) UMAP demonstrating integration of Nkx2-1 knockout AEP-O and WT AEP-O by source (left) or cell state (right). **(B)** Extent of Nkx2-1 KO specific open chromatin changes across the murine genome.

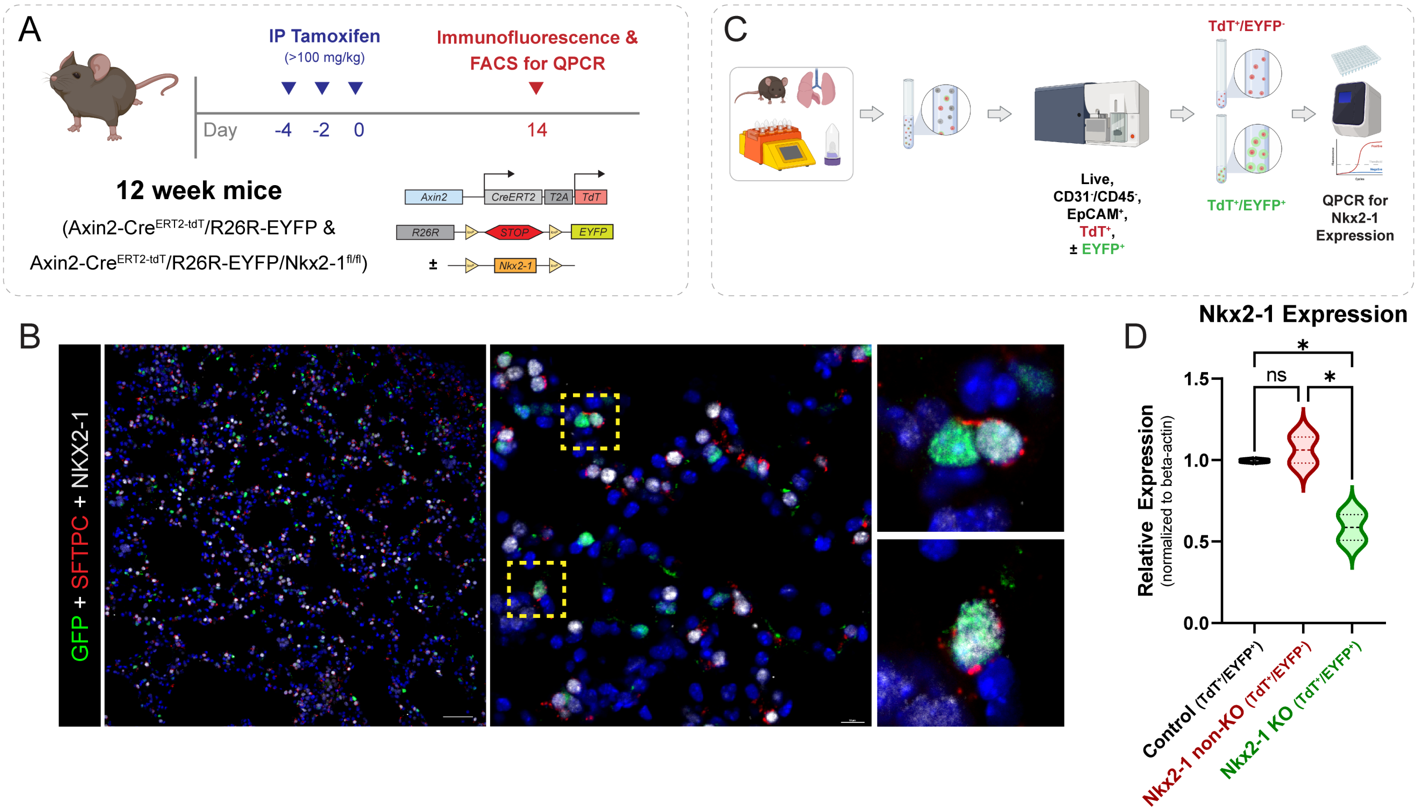

Supplemental Figure 12 | Efficiency of Axin2-CreERT2 in generating biallelic knockouts in the epithelium.
(A) Experimental design to generate Nkx2-1 null AEPs using $Axin2^{CreERT2}$. **(B)** IHC at 2 weeks post tamoxifen injection demonstrating lineage labeled AT2 cells expressing Nkx2-1 protein, suggesting incomplete knockout in the AEP lineage. **(C)** Experimental design to evaluate efficiency of Nkx2-1 knockout in lineage labeled cells. **(D)** Nkx2-1 expression was reduced by approximately 50% in lineage labeled cells, confirming inefficient recombination despite high dose tamoxifen via the $Axin2^{CreERT2}$. [*Scale bars = 50 μm and 10 μm*]. Source data are provided within the "Source Data" file. Schematics created with Biorender.com.

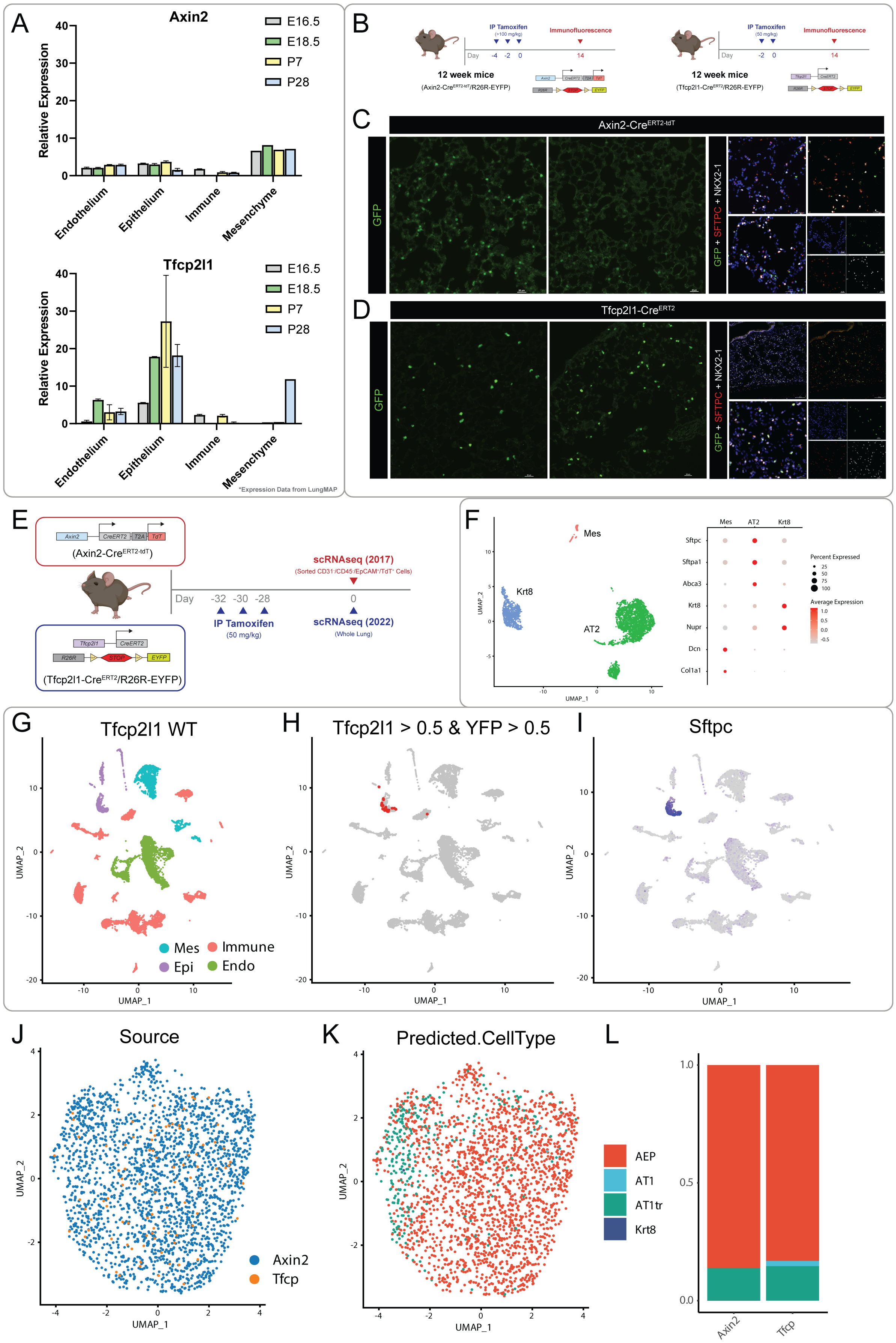

Supplemental Figure 13 | Tfcp2l1Cre^{ERT2} functions as an epithelial-specific method to target the AEP lineage.
(A) Comparison of expression level of Axin2 and Tfcp2l1 in published LungMAP data[79,80] shows significant epithelial enrichment. **(B-D)** Comparison of lineage labeling in homeostatic lung using Axin2^{CreERT2} and Tfcp2l1^{CreERT2}. **(E)** Experimental design to compare the molecular state of Tfcp2l1-lineage cells with Axin2$^{CreERT2\text{-}TdTomato}$ sorted AEPs. **(F)** scRNAseq of sorted AEPs, reproduced from Figure S5 for comparison. **(G-I)** UMAP project of Tfcp2l1^{CreERT2} x R26R^{EYFP} whole lung scRNAseq confirms Tfcp2l1-lineage cells comprise a subpopulation of AT2 cells in adult homeostatic lung. **(J-L)** Comparison of molecular state of freshly sorted Axin2+ AT2 cells (from F) and Tfcp2l1-lineage labeled AT2 cells (from **G-H**). Integration of these cells leads to clustering in a single cell population, and label transfer from AEP-O scRNAseq identifies >80% of cells as in the AEP state. [*Scale bars = 25 μm*]. Schematics created with Biorender.com.

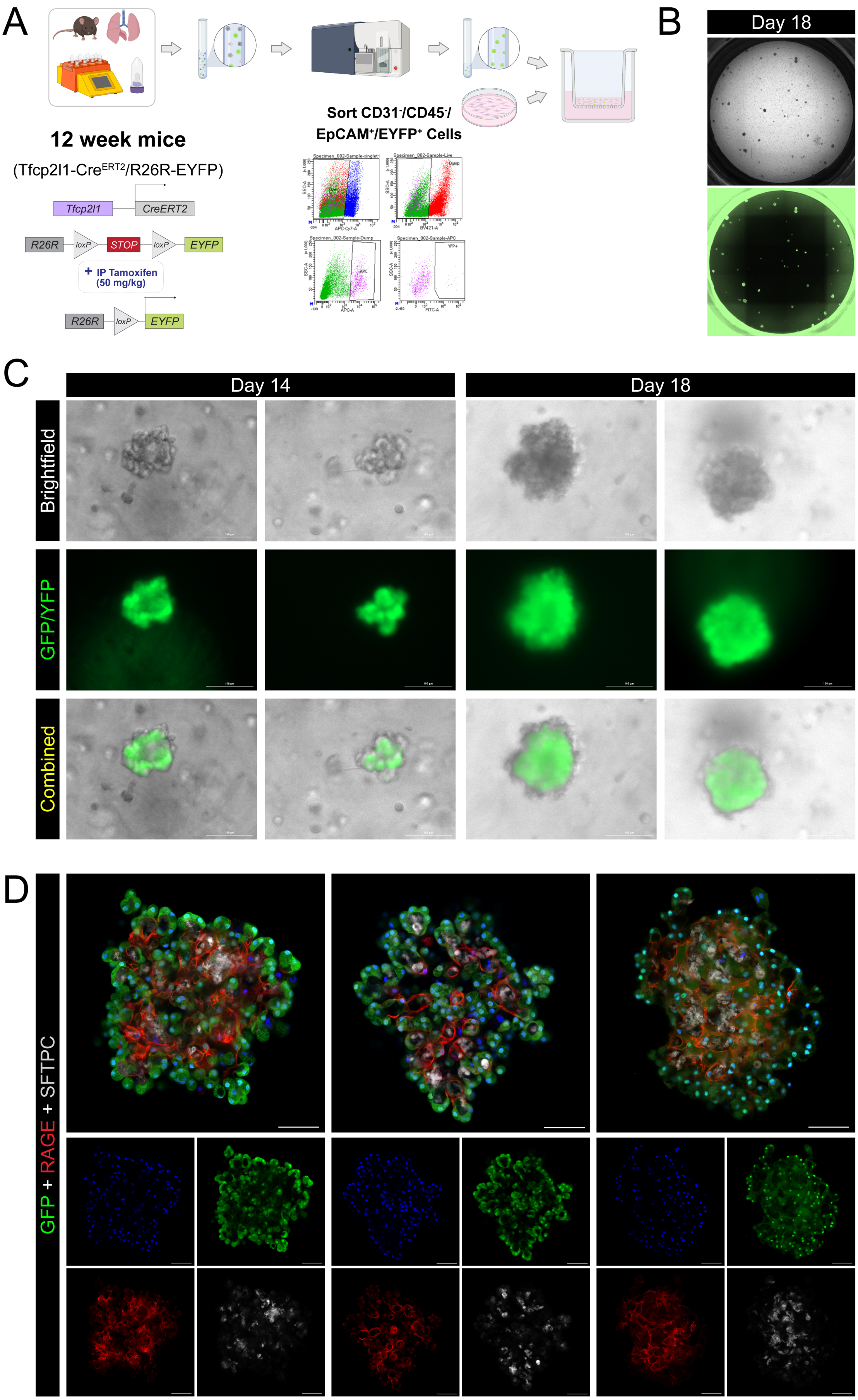

Supplemental Figure 14 | Tfcp2l1-lineage organoids form complex organoids in AEP-O culture conditions.
(A) Experimental design for generation of Tfcp2l1-lineage organoids. **(B)** Whole mount image showing virtually all organoids in culture derive from Tfcp2l1-lineage labeled EYFP+ cells. **(C-D)** Tfcp2l1-derived organoids form complex organoids which develop complex cellular differentiation and 3D organization indistinguishable from Axin2-lineage organoids. [*Scale bars = 50 µm*]. Schematics created with Biorender.com.

Supplemental Table 1a | Antibodies for Organoid Whole-Mount Immunofluorescence:

Primary Antibody (1:100 – unless otherwise specified)	Secondary Antibody (1:200)
**anti-Sftpc** (Guinea Pig, Seven Hills Bioreagents, GP992)	**Goat anti-Guinea Pig IgG Alexa Fluor 555** (Invitrogen, A21435)<br>**Goat anti-Guinea Pig IgG Alexa Fluor 647** (Invitrogen, A21450)
**anti-RAGE** (Rat, R&D Systems, MAB1179)	**Donkey anti-Rat IgG Alexa Fluor 488** (Invitrogen, A21208)<br>**Donkey anti-Rat IgG Alexa Fluor 568** (Abcam, ab175708)
**anti-TTF1/Nkx2-1** (Rabbit, Seven Hills Bioreagents, Rb1231)	**Donkey anti-Rabbit IgG Alexa Fluor 647** (Invitrogen, A31573)
**anti-Ki67** (Rabbit, Abcam, ab16667 [SP6])	**Donkey anti-Rabbit IgG Alexa Fluor 647** (Invitrogen, A31573)
**anti-GFP** (Chicken, Abcam, ab13970)	**Goat anti-Chicken IgG Alexa Fluor 488** (Invitrogen, A11039)
**anti-CD45** (Rat, BioLegend, 103103)	**Rabbit anti-Rat IgG Alexa Fluor 488** (Invitrogen, A21210)
**anti-E-Cadherin** (Rat, R&D Systems, MAB7481 – 1:250)	**Donkey anti-Rat IgG Alexa Fluor 568** (Abcam, ab175708)
**anti-Krt8** (Rat, DSHB, TROMA-I – 1:50)	**Donkey anti-Rat IgG Alexa Fluor 568** (Abcam, ab175708)

Supplemental Table 1b | Antibodies for Immunofluorescence on Paraffin Sections:

Primary Antibody	Secondary Detection
**anti-GFP** (Rabbit, Abcam, ab290 – 1:1000)<br>**anti-RAGE** (Rat, R&D Systems, MAB1179 – 1:100)<br>**anti-Sox2** (Rabbit, Seven Hills Bioreagents, Rb1236 – 1:200)<br>**anti-Sox9** (Rabbit, Sigma Aldrich, AB5535 – 1:100)<br>**anti-Cdx2** (Rabbit, Cell Marque, EPR2764Y – 1:500)<br>**anti-Gata4** (Rabbit, Santa Cruz, sc-9053 – 1:200)<br>**anti-Pdx1** (Goat, Abcam, ab47383 – 1:5000)	**TSA Plus Fluorescein** (Akoya Biosciences, NEL741001KT)
**anti-Krt8** (Rat, DSHB, TROMA-I – 1:200)<br>**anti-Sftpc** (Rabbit, Seven Hills Bioreagents, Rb09337 – 1:250)	**TSA Plus Cyanine 3.5** (Akoya Biosciences, NEL763001KT
**anti-Ki67** (Rabbit, Abcam, ab16667 [SP6] – 1:100)<br>**anti-E-Cadherin** (Rat, R&D Systems, MAB7481 – 1:1000)	**TSA Plus Cyanine 3.5** (Akoya Biosciences, NEL763001KT)<br>**TSA Plus Cyanine 5** (Akoya Biosciences, NEL745001KT)
**anti-TTF1/Nkx2-1** (Rabbit, Seven Hills Bioreagents, Rb1231 – 1:1000)	**TSA Plus Fluorescein** (Akoya Biosciences, NEL741001KT)<br>**TSA Plus Cyanine 5** (Akoya Biosciences, NEL745001KT)

Supplemental Table 2 – scRNAseq Output Summary:

Dataset	Organoid Day 14	Organoid Day 21	Organoid Day 28	Organoid AAV Control	Organoid Nkx KO	Tfcp2l1 Lung EYFP Control	Tfcp2l1 Lung Nkx KO
**Estimated Number of Cells**	9,908	9,519	5,546	9,184	12,694	12,259	13,268
**Mean Reads per Cell**	40,509	42,230	69,615	63,819	38,811	29,115	32,067
**Median Genes per Cell**	3,606	3,637	4,431	2,766	2,452	1,741	1,686
**Number of Reads**	401,364,390	401,993,978	386,086,102	586,113,691	492,668,484	356,924,215	425,460,902
**Valid Barcodes**	97.90%	98.20%	98.20%	97.60%	97.70%	96.70%	96.30%
**Sequencing Saturation**	23.40%	28.90%	41.90%	28.60%	28.00%	68.00%	63.00%
**Q30 Bases in Barcode**	95.80%	95.80%	95.80%	96.70%	96.70%	97.10%	97.10%
**Q30 Bases in RNA Read**	93.30%	93.60%	93.70%	94.20%	93.90%	95.40%	95.60%
**Q30 Bases in UMI**	95.40%	95.40%	95.40%	96.20%	96.20%	97.10%	97.20%
**Reads Mapped to Genome**	90.90%	91.90%	93.00%	82.80%	85.60%	90.20%	88.90%
**Reads Mapped Confidently to Genome**	86.70%	88.00%	89.20%	78.30%	82.30%	84.50%	82.70%
**Reads Mapped Confidently to Intergenic Regions**	6.00%	6.10%	7.20%	8.50%	9.30%	7.60%	10.30%
**Reads Mapped Confidently to Intronic Regions**	12.70%	11.10%	10.40%	11.80%	10.70%	14.40%	16.80%
**Reads Mapped Confidently to Exonic Regions**	67.90%	70.70%	71.60%	58.00%	62.30%	62.50%	55.50%
**Reads Mapped Confidently to Transcriptome**	64.50%	67.70%	68.80%	55.20%	60.00%	69.70%	64.70%
**Reads Mapped Antisense to Gene**	2.00%	1.60%	1.40%	1.90%	1.30%	6.90%	7.40%
**Fraction Reads in Cells**	76.70%	78.20%	83.70%	48.90%	83.70%	83.40%	78.30%
**Total Genes Detected**	20,300	20,134	19,308	22,830	22,952	26,904	29,411
**Median UMI Counts per Cell**	13,384	14,538	22,287	9,140	8,148	3,850	3,690

Supplemental Table 3 – scATACseq Output Summary – v1 assays:

Sample ID	Organoid Day 14	Organoid Day 21	Organoid Day 28
Annotated Cells	13,300	11,765	4,535
bc_q30 Bases Percent	89.4%	90.0%	78.4%
Cellranger-ATAC Version	1.2.0	1.2.0	1.2.0
Cells Detected	13,300	11,765	4,535
Percent Cut Fragments in Peaks	47.0%	34.7%	60.3%
Percent Fragments nfr	67.8%	71.0%	68.6%
Percent Fragments nfr or nuc	94.4%	96.1%	93.2%
Percent Fragments nuc	26.6%	25.1%	24.6%
Percent Fragments Overlapping Peaks	49.1%	36.7%	62.8%
Percent Fragments Overlapping Targets	63.4%	55.6%	74.6%
Percent Mapped Confidently	79.3%	76.9%	69.3%
Percent Waste Chimeric	0.6%	0.4%	0.5%
Percent Waste Duplicate	13.6%	7.7%	10.6%
Percent Waste lowmapq	5.2%	4.0%	3.4%
Percent Waste Mitochondrial	0.3%	0.3%	0.7%
Percent Waste No Barcode	1.7%	1.7%	5.7%
Percent Waste Non Cell Barcode	46.6%	66.9%	54.0%
Percent Waste Overall Nondup	55.1%	73.6%	66.0%
Percent Waste Total	68.7%	81.3%	76.6%
Percent Waste Unmapped	0.7%	0.4%	1.6%
Median Fragments Per Cell	10,746	7,700	13,310
Median per Cell Unique Fragments at 30000 RRPC	4,135.0	2,578.2	4,064.0
Median per Cell Unique Fragments at 50000 RRPC	6,501.4	4,095.3	6,437.5
Num_Fragments	6.13E+08	6.26E+08	2.73E+08
r1_q30 Bases Percent	94.4%	94.3%	86.6%
r2_q30 Bases Percent	94.0%	94.0%	85.2%
si_q30 Bases Percent	92.4%	92.5%	76.4%
Total Usable Fragments	1.92E+08	1.17E+08	6.40E+07
TSS Enrichment Score	6.360	4.651	8.864

Supplemental Table 4 – scATACseq Output Summary – v2 assays:

Sample ID	NKX-KO-Org-ATAC	Tfcp2l1-Ctrl-Lung-ATAC	Tfcp2l1-NKX-Lung-KO-ATAC
Cellranger-ATAC Version	2.00	2.0.0	2.0.0
Estimated number of cells	16748	9079	11655
Confidently mapped read pairs	0.8697	0.909	0.9066
Estimated bulk library complexity	5.00E+08	5.01E+08	4.83E+08
Fraction of genome in peaks	0.06	0.0595	0.0585
Fraction of high-quality fragments in cells	0.6842	0.9268	0.8974
Fraction of high-quality fragments overlapping TSS	0.3619	0.4116	0.4177
Fraction of high-quality fragments overlapping peaks	0.6804	0.6534	0.6657
Fraction of transposition events in peaks in cells	0.6459	0.623	0.6372
Fragments flanking a single nucleosome	0.281	0.3241	0.2851
Fragments in nucleosome-free regions	0.608	0.5951	0.639
Mean raw read pairs per cell	31246.8	40285.46	33149.51
Median high-quality fragments per cell	9195	20816	15064
Non-nuclear read pairs	0.021	0.0041	0.0017
Number of peaks	189011	192042	187335
Percent duplicates	0.3786	0.3085	0.3312
Q30 bases in barcode	0.884	0.8704	0.8724
Q30 bases in read 1	0.9544	0.9317	0.9335
Q30 bases in read 2	0.9395	0.9158	0.9189
Q30 bases in sample index i1	0.9277	0.8875	0.8904
Sequenced read pairs	5.23E+08	3.66E+08	3.86E+08
Sequencing saturation	0.5244	0.4336	0.4598
TSS enrichment score	12.3737	14.5047	15.7765
Unmapped read pairs	0.0175	0.017	0.0148
Valid barcodes	0.9575	0.9551	0.9545
